# Re-inventing care planning in mental health: stakeholder accounts of the imagined implementation of a user/carer involved intervention

**DOI:** 10.1186/s12913-015-1154-z

**Published:** 2015-10-30

**Authors:** Helen Brooks, Caroline Sanders, Karina Lovell, Claire Fraser, Anne Rogers

**Affiliations:** EQUIP, School of Nursing, Midwifery and Social Work, University of Manchester, Oxford Road, Manchester, M13 9PL UK; Centre for Primary Care, Institute of Population Health, The University of Manchester, Williamson Building, Oxford Road, Manchester, M13 9PL UK; NIHR CLAHRC Wessex, Faculty of Health Sciences, University of Southampton, Highfield, Southampton, SO17 1BJ UK

**Keywords:** Implementation, Normalisation process theory, Qualitative, Mental health, Health services

## Abstract

**Background:**

Despite an increase in mental health innovations designed to increase service user and carer involvement in services, there is evidence that service users and carers are still relatively marginalised. This study aimed to identify key informants operating with knowledge of both policy and practice related to future models of mental health management in order to explore the potential de-implementation of existing care planning and possibilities for the introduction of a training programme designed to implement a new user and carer involved and focussed process of mental health care planning.

**Methods:**

13 semi-structured interviews were carried out with key informants from a range of relevant disciplinary backgrounds and professional roles, who were involved locally and nationally in policy, practice and research. The aim of the interviews was to explore their perspectives on contemporary arrangements for care planning procedures and processes and to identify factors that might promote or inhibit the routine incorporation of user/carer led planning. Findings were compared to data derived from service users, carers and professionals to illuminate added value.

**Results:**

Key stakeholders identified elements of the current care planning context that were likely to impact on the implementability of user - focussed care planning. Like other stakeholders, key informants felt that the proposed intervention coalesced with the increasing normalisation of user involvement as appropriate and desirable. Participants added to existing data by illuminating the need for organisational bureaucracy and the legacy of prior mental health policy and historical practice to be considered in implementation. Adequate relationships within the system were considered by all stakeholders to be crucial to successful implementation and key informants discussed how this could be eroded through attempts at practice standardisation and current connectivity and culture within services.

**Conclusions:**

The study demonstrated the value of incorporating the perspective of stakeholders not directly involved in service delivery in implementation research designed to inform an intervention at the point of design. Their contribution centred on the identification of factors that appeared not be obvious to those working in the system or emanated from political and policy arenas as well as developing the contextual understanding of themes raised by other stakeholders.

**Electronic supplementary material:**

The online version of this article (doi:10.1186/s12913-015-1154-z) contains supplementary material, which is available to authorized users.

## Background

The last decade has witnessed a proliferation of innovations in mental health including a rapid growth in user-based models which seek to change the way patients or users interact with service providers [1]. This has occurred at a time of social and technological change and in a context of a growing recognition of the need to act on the marginalised status of mental health provision [2]. How mental health innovations are implemented and their relative success of being embedded and sustained has also been a focus for current research [3]. Recently, mental health innovations have included user-led and recovery-oriented models, which have been predicated on the possibilities of service users taking increasing control of their lives. This focus has orientated commissioners and providers of services to the potential for developing interventions that focus on service user and carer experience. One area where a wish for change has been articulated by service users is in relation to care planning [4, 5]. The NHS Choices website defines a care plan as an agreement between a service user and their health professional designed to help them manage their health day to day. Mental health policy documents, reviews of best practice and literature produced by user and carer groups advocate the involvement of users and carers in care planning as a means of improving the quality of care and promoting recovery [6, 7]. However, there is substantive evidence that this does not occur spontaneously and requires dedicated attention and action at a variety of levels [4, 5].

Enhancing the Quality of User Involved Care Planning in Mental Health Services (EQUIP) is designed as a programme of work which aims to improve service user and carer involvement in care planning. Users and carers report feeling excluded, unsupported and distanced by mental health services and want much more involvement in the care planning process [4, 5, 8, 9]. EQUIP aims to improve user and carer involvement in care planning in mental health services through the development of a training package for health professionals so that they can enhance service user and carer involvement in their care. The training will be developed and delivered by users/carers, researchers and health professionals for multiple mental health and social care professionals within Community Mental Health Teams (CMHTs).

This study sought to inform the process of developing the EQUIP programme by presenting stakeholder (in this case, key informants within health care and academic domains of mental health expertise) views of care planning, and expectations of individual and organisational ‘barriers’ and ‘facilitators’ to user/carer involved care planning. Whilst accounts from those involved with the delivery of services is relevant and informative, it may lack the dimensions likely to be illuminated by stakeholders best able to identify a range of factors that may not be obvious to those working in the system or emanate from political and policy arenas. What currently remains relatively hidden from view is how the environment of those in receipt of mental health services and management is shaped by interests and influences of a wide range of micro- and meso- level influences. Thus our aim here is to rebalance the focus on the micro individual action orientation of those operating within individual service contexts through tapping the hidden but relevant influences of institutional organisational arrangements and meso-level influences of political/economic policy through examining the perspectives of other key stakeholders. Data will be compared to that obtained from service users, carers and mental health professionals already presented elsewhere [10–12].

### Thinking about implementation

Normalisation Process Theory (NPT) has been used to consider complex interventions prior to the development of a randomised control trial to test their effectiveness [13]. It has also been used in the context of mental health to explore the impact of new forms of collaborative care on the way in which professionals carry out their routines of work in primary care [14]. The four constructs (coherence, cognitive participation, collective action and reflexive monitoring) permit a means of appraising factors that might ‘promote and inhibit the routine incorporation of complex interventions into everyday life’ ([13], pp.64). It focuses on the work that people need to do to ensure interventions become ‘normalised’. As a heuristic framework it can support the optimisation of a trial intervention at three points:

- supporting intervention design
- describing the context of a trial
- supporting the interpretation of a trial’s results

**Fig. 1:**
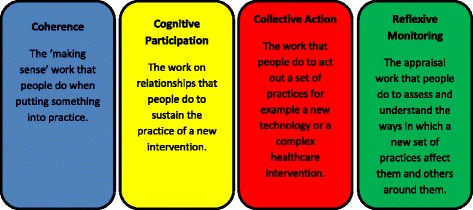
Description of NPT components

At the outset we used NPT in terms of *“thinking about the doing”,* that is, making sense of the options regarding the design of user/carer led care planning training (see Fig. 1). The two constructs which had most relevance were *coherence* and *cognitive participation* which focus on the underlying meaning of the phenomenon being studied and how a new practice is mobilised (see Fig. 1). This contrasts with the desirable use of NPT at a later point when assessing implementation which is more closely associated with the construct of collective action (the basis of the model) and reflexive monitoring [15]. These constructs focus more specifically on how a new intervention/aspect of practice is mobilised. Given that this study asked participants to consider NPT at the pre-design/training level and reflexive monitoring is more pertinent to looking back and reflecting on implemented practice, this component will not be a focus of the current paper.

### Aim of the study

In the context of predicting the likely implementability of user/carer involved care planning, this study sought to understand key informant perspectives on contemporary arrangements for care planning procedures and processes by:

- exploring the potential facilitators and barriers to the implementation of user/carer led planning within health services.
- using NPT to identify factors that might promote or inhibit the routine incorporation of user/carer led planning with a particular focus on coherence and cognitive participation.

## Methods

The key informants interviewed included respondents from a range of relevant disciplinary backgrounds and professional roles who were involved locally and nationally in policy, practice and research. It was envisaged that key informants would have access to insider policy and practice information, diverse relationships and opinions. This knowledge is likely to be important to understanding potential implementation issues prior to the undertaking of a clinical trial which will test the effectiveness of a training package for mental health professionals designed to improve service user/carer involvement in care planning. We used the NPT toolkit framed as a set of propositions to guide the stakeholder interviews [16]. For example, questions included those such as ‘how do you think participants would distinguish between a user led approach and current care planning?’ and ‘what would you see as being the purpose of the user led care planning approach?’. The interviews used a few open questions to trigger responses, followed by questions to clarify or illuminate specific items.

Potential interviewees were sent an email with a participant information sheet and consent form explaining the background to the study and what participation would entail. Interviews were carried out face to face or via the telephone between January and June 2014. Participants were offered a choice of interview at a time and date convenient to them. The method employed depended on participant preference and the distance from the project base. Interviews lasted between 35 min and an hour and a half and were digitally recorded before being transcribed verbatim by an external transcription company. A particular focus of the interviews was on the feasibility of delivering a user/carer led training package to improve user/carer involvement in care planning (see Additional file 1 for an interview schedule). This feasibility related specifically to:

- understanding user involvement and participation
- multidisciplinary perspectives
- knowledge and experience of care planning
- resource allocation
- official policy catalysts
- organisational cultural acceptance
- organisation resistance and facilitation

Transcripts were returned to the research team who anonymised them and allocated them for analysis.

The study also included a mapping exercise of organisational structures and policies related to care planning which was reviewed and updated over the course of the project at the two geographical areas to be included in the trial. The aim of this component was to understand the pre-existing arrangements and views of care planning operating at differing organisational levels by:

- analysing trust documents relevant to care planning
- exploring audits related to care planning
- identifying how care planning activities are recorded
- reviewing relevant service user experience from surveys/committe min/audits relating to care planning

Two brief reports of findings were produced from relevant documents/policy/stakeholder groups, national and local survey results and complaints/compliments relevant to care planning. In addition, members of the wider EQUIP team met to discuss and look at relevant policies/procedures and documentation relevant to care planning (i.e. Care Planning Approach).

Members of the team also examined the IT systems utilised at each site (to record care planning) and were provided with a selection of (excellent and poor) anonymised care plans.

### Sample

Stakeholder interviews were considered relevant for illuminating the range of perspectives of a new or existing policy innovation's value in addition to that of service users, carers and front line health professionals reported elsewhere [10–12]. It was relevant to obtain such meso- level views in the current context given the proliferation and range of user involved innovations and the complexity of the mental health field.

Participants from prominent knowledge positions were recruited. These included senior or executive level NHS managers and key national academics and policy makers. These individuals were selected on the basis that they were likely to be immersed in critical understanding of contemporary care planning and user involvement. The individuals who made up the sample were identified through purposive and snow-balling techniques starting from a list of national key contacts developed by the authors. We asked the respondents interviewed for names of other potential interviewees. Sampling occurred in this manner until 13 interviewees were recruited (see Table 1). Each participant was expected to have knowledge, which was not held by others, thus we were not necessarily expecting saturation in the interviews.

**Table 1:** Demographic information relating to study participants

	Number
Gender	
Female	7
Male	6
Total	13
Employment	
Within services	4
Within academia	9
Total	13
Method of interview	
Face to face	1
Phone	12
Total	13

As part of the wider EQUIP project, focus groups and interviews were carried out with a wide range of service users, carers and health professionals (see Table 2).

**Table 2:** Number of service users, carers and professionals included in wider EQUIP study and used for comparison

	Focus groups	Interviews	Total
Service user	24	22	46
Carer	11	26	37
Service user/carer	3	1	4
Professional	23	28	51

### Analysis

In line with other studies, data was analysed using the NPT constructs as a set of sensitising concepts for carrying out the study [17, 18] and for analysing the data once collected combined with thematic analysis [19]. Thematic analysis identifies, analyses and reports themes occurring in a dataset. For the purposes of this study, this was a set of interview transcripts. A summary descriptive diagram detailing each component of the NPT can be found in Fig. 1.

For the first stage of analysis, the transcripts were read and the text assigned initial codes by two of three independent researchers (HB, AR, CS). Similar codes were then amalgamated into themes. The authors also examined data that could not be incorporated into the framework so that important aspects of the data were not excluded. After the first coding round, authors (HB, AR, CS) met to discuss the coding framework and emerging themes. This process involved clustering identified themes into high-ranking themes and deleting redundant themes. During this meeting, deviant cases and any codes that fell outside of the framework were also discussed.

Thematic analysis of interview data and snowball sampling were conducted according to the constant comparative method. Analysis was carried out concurrently with data collection and sampling so that emerging issues could be explored iteratively. Once consensus was reached, the first author reanalysed the transcripts based on the revised framework and drafted the paper. All co-authors commented on and added to this and subsequent drafts to produce a final manuscript.

The analysis process was managed using an Excel spreadsheet, which included demographic information about study participants. The emerging framework was saved in its various iterations in word documents for the purposes of transparency and to provide an audit trail.

Regular meetings between the study team and the wider research team enhanced the trustworthiness of the data by ensuring the emerging codes remained grounded in the data, and provided the opportunity to discuss alternative explanations. In addition, the emerging framework was presented to the wider EQUIP team (including service user and carer representatives) not involved directly in data analysis, who were then asked to critically comment on the developing framework in terms of ambiguities, omissions and clarity.

### Ethical approval

Ethical approval was sought and obtained from the University of Manchester’s Research Ethics Committee in 2014, reference number 13304.

## Results

The results are presented under the seven main emergent themes, which arose during the process of analysis (Additional file 2 details from which NPT component these themes were derived from). These included shifting the ownership of care plans, bridging the translational gap – the role of bureaucracy and historical context, the importance of relationality within the system, the value and nature of the work associated with care planning, the role of the individual versus the collective within health services, individual differences and the impact of organisational culture. Data is provided from the key informant interviews to demonstrate the added value of including their voice in the narrative about potential implementation threats.

### ‘Shifting ownership’ - understanding the need for empowerment and person-centred care planning

Key informants indicated the presence of a sense of coherence about what ‘good’ current care planning should entail. There was also a shared understanding of what needed to change to be able to implement successful care planning focussed on user experiences which echoed the views of service users, carers and professionals found in the wider EQUIP study [10–12]. Participants in the current study considered that care planning produces optimal outcomes when service users feel in control of their care and linked this to the personalisation agenda - the importance of choice shifting the balance of power in relationships with health professionals and challenging traditional, paternalistic models of health care delivery. To illustrate, one participant stated care plans work best when they are ‘*completely written with the service user and are geared around their needs*’ **Key Informant 2 (Female, Manager, NHS).** The following participant demonstrates how optimal care planning involves collaboration and working together with the service user to achieve their goals.

*‘I guess that’s for me what care planning is. Each step of the way it’s taking an immediate kind of measure of… It’s not a history, it’s what are your problems now? How do we help you to overcome them and how do we help to build the goals to, you know, getting out of hospital then getting, you know, getting kind of the lessons you’ve learned here into your everyday life’.***Key informant 8, (Female, Professor, Academia).**

### Bridging the translational gap – the impact of bureaucracy and the history of control and coercion within services

Following the data collected in the wider EQUIP study from service users, carers and health professionals, key informants also felt that care planning policies often failed to translate into practice. The data from key informants helped illuminate some of the potential reasons cited for this translational gap which included: the historical context of care planning, differences in individual staff members’ skills and experience and a lack of a shared definition of care planning.

Participants spoke about the historical context of care planning and how they felt it arose from concerns about risk management. Consequently, care planning was associated with strong links to coercive aspects of the Mental Health Act and a lasting agenda about control.

*'… because of the context of risk management, particularly in the coercive context, where you’ve got a mental health act, I think it inevitably colours, really, how people think about it, what they do with it, what they prioritise. Um, so in a sense…you know, I can’t answer whether care planning is a good or bad thing in the abstract, without putting it into the context of, why was it used, and the fact that there’s a legal framework for it.’***Key informant 9 (Male, Professor, Academia).**

Competing drivers and understandings amongst different stakeholder groups meant that there was an apparent lack of a shared definition of care planning leading to variation in delivery and in some cases low levels of involvement in care planning.

*‘All the policies of involvement [are] great, and this is what should be happening, but in practice, it wasn’t really happening…*

*…But that was one of the problems, if you look into it, was that nobody really knew what it looks like. What is involvement, you know?*

*…It’s a very easy word for people to use, like participation and all the rest of it. But, in fact, in clinical practice, well in any situation, nobody really knows what it looks like.*' **Key informant 7 (Female, Post-Doctoral Researcher, Academic).**

The complexity of the matters being addressed by the formalised care planning process was identified in both the interviews and the mapping exercise. This was viewed by key informants as being exacerbated by a policy of care planning being introduced with insufficient explanation to either professionals or service users. This indicated there has been a lack of coherence from the inception of care planning. One such complexity was the prioritisation of safety which went against the grain of the shared understanding around the need for user-centred approaches to care planning. This may go some way to understanding the lack of a shared definition of care planning identified by front line professionals [10].

*'And it was, erm…and it was really interesting, because I think every care plan basically stated five goals…*

*…you know, or five objectives, and they were things like, if I can remember, they were things like offer one to one, but there was no, sort of, you know, explanation of what that one to one would consist of…*

*…or what would be the purpose of it. It would be, eh, maintain safety. That was another one.*

*…But there was no actual descriptors of what maintaining safety would mean.'***Key informant 6 (Male, Senior Staff, Department of Health).**

Some participants felt that involvement in care planning worked better **prior** to the introduction of policies designed to formalise the process. Whilst participants acknowledged that policies were designed with good intention, attempts to standardise the process meant that there was limited flexibility and this coupled with the associated bureaucracy served to actually reduce the levels of involvement across services. Care planning subsumes the service user to the requirements of the bureaucratic process, thus undermining what might have been deemed as user-centred care and shifting it to a more bureaucratic - centred service provision. Often this was because policy introduced a ‘lowest common denominator’ effect which was perceived to lower standards. Additionally, whilst ‘bureaucracy’ has the potential to raise the profile of care planning within Trusts, it also means involvement is not seen as a long-term process with social and health outcomes and instead is broken down into events (e.g. inpatient episodes). This episodic approach encouraged by data collection and monitoring requirements reduced continuity (seen as important for care planning) and is seen as further exacerbated by fragmentation of services and roles within services.

*‘So, I think in terms of the, sort of, procedural things the problem is it’s something that’s quite often well intentioned, or sadly based, or evidenced based, like a, sort of, care programme approach, you know, the care programme review meeting, or something, eh, could actually deteriorate into a, sort of, stereotyped, sort of, erm, almost a mockery of participation in practice.’***Key informant 5 (Male, Professor, Academia).**

*‘People not being quite sure exactly what it is they want, you know, the whole realities of working with a human being as opposed to this kind of in a way bureaucratic process which is designed to chart and monitor.’***Key informant 11 (Male, Post-Doctoral Researcher, Academia).**

### Sufficient relationality within the system is more important than practice innovations

Participants often felt that it was the relationship between the service user and professional that was more useful that the care plan itself and it was actually these relational aspects that needed improving across services, which echoed findings from the wider EQUIP study. However, this was difficult because the therapeutic relationship and associated complexities were difficult to translate into policy and associated guidelines and relied instead on individual, interpersonal skills.

*‘A lot of it comes down to how you…how you relate to people and the kind of relationships you try and build with your patients and… so I do think an awful lot of this is about…it’s not just about, you know, the [] kind of physical thing about doing the care plan. It’s about professional patient service user relationships.’***Key informant 12 (Female, Professor, Academia).**

Data from front line professionals alluded to senior management being too far removed from front line services [10]. This was echoed in the data from key informants within health services which demonstrated that the more senior the health professionals were, the more likely they were to consider that care planning was done well within the Trust, which contradicts the knowledge and information from front line staff and service users/carers [4, 5, 8, 9]. This illustrates that there are multiple levels of constructed coherence within a mental health system. There may be a sense of coherence at a senior level that differs from shared understanding or lack of coherence) at the level of practice. This could become an impediment to enabling user involved care planning to work in practice via collaboration between the actors at multiple levels.

Some participants acknowledged this distance of the operational focus of higher level management from day to day services which meant they were perceived as not understanding the everyday barriers and difficulties in involving service users and carers and undertaking care planning in a collaborative way.

*‘At the top level, they’re the…they’re the people you should take least notice of.*

*It’s like the stuff on recovery, you know, if you look at a website of a mental health trust, you think that all they ever did was do recovery plans for patients…*

*…You wouldn’t think the mental health act existed, you know, it’s just all bollocks.’***Key informant 9.(Male, Professor, Academia).**

### The value of and nature of work associated with implementing user/carer involved care planning

In line with service users, carers and front line professionals, key informants could see the value of user and carer involvement in care planning. Where it worked well it was seen to be associated with positive outcomes.

*‘Well, they’re essential in it, because it’s their care plan and they’re the mental health service user, so they should be absolutely intrinsically involved.’***Key informant 3 (Female, Senior Manager, NHS).**

The meso level of understanding provided by including the key informants highlighted the wider contextual issues along with the nature of the work required to implement user and carer involved care planning. Care planning was viewed as a useful recording process and platform to raise matters that would not have been possible to raise via other means. Respondents felt that implementing and sustaining user and carer involved care planning required “hard work” or cognitive participation by all parties. This needed to be combined with a culture of involvement within services, support for staff and the testing of the degree of cultural adaptation over time.

*'We have a National Institute for Clinical Excellence, what I think we need is a national institute for management excellence so that we support middle and higher manage…management and I mean, clinical managers as well as kind of service managers that actually understand the evidence based practice or the evidence based methods for driving implementation. But there’s no…there is no short cut to having it in a culture at the organisation, making sure at induction everybody knows it’s a key priority.'***Key informant 10 (Female, Senior Staff, Department of Health).**

### The individual versus the collective and the inhospitable ethos of mental health services

Key informants predicated a degree of ambivalence towards doing the work required to sustain user/carer involved care planning because of historical context factors. A lack of engagement amongst service users might result from prioritising an ethos of personalisation over the collective efficacy characterising the orientation of service user group action.

*‘I guess the other contention around care planning, is the fact it’s individualised, and a lot of the debates around the quality of mental health services are collective. And I can give a good example of this… the contract formation of individuals compared to whether or not a service is being provided for the collective’.***Key informant 9(Male, Professor, Academia).**

The use of a formulaic and restrictive, ‘tick box’ type approach (also identified via the mapping exercise) could militate against involving service users and carers in care planning. The prioritising of ‘Paperwork’ was seen as a barrier to involvement and there was a perception that most actual involvement work goes on outside of formal meetings in the absence of formal documentation.

*‘In terms of how you involve people and what actually happens in that sort of therapeutic relationship level. That’s not really something that is necessarily looked at.*

*And it’s also not necessarily something that’s supported. It’s more about, have you ticked all the boxes? Have you done the right paperwork? Have you, you know, all that sort of thing…*

*…Rather than, how did you actually relate to the person you were talking to?’***Key informant 7 (Female, Post-Doctoral Researcher, Academia).**

*'So it’s kind of you use the documentation to give a very broad stroke framework and then the meat of it, the actual meeting is what you do which is, if you like, the engine of it, is what you actually do in the meetings. I mean, one it’s so the patient isn’t too daunted by the paperwork. Paperwork can get in the way.’***Key informant 11 (Male, Post-Doctoral Researcher, Academia).**

In line with this, a perception was evident amongst key informants that formal care planning documentation has historically been associated with a legal imperative to monitor practice. However, the reality of practice focussed more on choice and individual relationships with professionals, which is not monitored or evaluated in the same way. Again, care planning was seen as something that should be central to all work within services, as well as relationships within services and not just at care planning meetings or with care planning documentation.

### The role of individual differences in relation to the manner of the delivery of user carer involved care planning

Some key informants felt that at times care co-ordinators felt disempowered or lacked confidence in engaging users and carers with care planning specifically as well as wider services more generally. This echoed the data collected from health professionals themselves in the wider EQUIP study. Reasons for this included communication difficulties (e.g. staff not speaking the same language as the service user), lack of expertise (particularly with physical health concerns), concerns about confidentiality and maintenance of disparate power dynamics in relationships with service users and carers. Again, participants talked about how paperwork and processes bore little relation to practice and made outcomes inaccessible and were often inappropriate.

*‘So some of their skills are clearly better in other areas…in some bits than other areas and I don’t think exactly anybody is an expert in all of it, so we’ve got to work much more as a team to make sure they pull on other members of the team that might be at certain elements of it.’***Key informant 1 (Male, Senior Manager, NHS).**

General Practitioners were seen as critical to the process but were often not invited or did not attend care planning meetings. The fact that key stakeholders are commonly absent clearly detracted from the capacity for necessary shared practice.

*‘I think for me one of the things that was often lacking was the fact that, you know, it’s the GP that’s often the person who’s most immediately involved in managing a crisis out in the community, very often…*

*…Umm, certainly where I worked, you know, the GPs were quite involved, probably more so than in a city area, but we didn’t get them along to the CPA meeting, because usually we held the CPA meeting in the hospital.’***Key informant 12 (Female, Professor, Academia).**

### Connection and culture as barriers to implementation

Continuity between services was seen as critical to the genesis of a meaningful process of care planning. Key informants often talked about a lack of connection and communication between inpatient and outpatient care. Health services, generally, were viewed as being in a state of constant flux operating in a context of limited resources which made continuity of care and choice difficult.

*‘Part of the difficulty we have now in the way that mental health services have been organised is that in some places you’ve now got a different care team looking after them as in-patients than you have as out-patients, because the consultants have been split.’***Key informant 12 (Female, Professor, Academia).**

*‘It [the paperwork] changes all the time, and they’re constantly having to look at a new system and how that works, which makes life really hard…*

*… It’s very difficult to have a discussion around what do you want, and how do you see things moving forward, how can we work on this, if in fact, your options are pretty limited.’***Key informant 7 (Female, Post-Doctoral Researcher, Academia).**

Current organisational culture was seen as an inhibitor to care planning; examples of this included pressure to reduce workload (through discharging service users from services), high turnover of staff and fragmentation of services. Sufficient time was viewed as an organisational barrier. Key informants felt that staff had less time than they used to to spend with service users given the limited resources. This was exacerbated by the amount of paperwork and associated bureaucracy which was often not factored into workload. Inpatient services were still perceived to have a focus on beds and the use agency staff, which further impacted on the time staff could spend with service users and subsequently on continuity and involvement. Targets within host organisations identified through the mapping exercise often referred to quantitative matters (e.g. does service user have a copy of the care plan? yes/no) rather than focussing on the quality of care plans.

*‘A factor that’s a real barrier for that is the turnover of staff within teams. People coming and going, temporary locums, and bank nurses, and so on, coming to post, so they, actually, may have a contact with the team, but there’s no individual they’d see more than perhaps for a few weeks, or a few months at a time.’***Key informant 5(Male, Professor, Academia).**

*‘So the official thing about the box ticked, how many people on the care programme approach, according to the statistics they send to the Department of Health, it’s all there in numbers but it’s meaningless.'***Key informant 9 (Male, Professor, Academia).**

Key informants added to the data provided by service users, carers and professionals through their contribution to the understanding of the meso-level context in which care planning operated. For example, key informants felt that Trusts were designed to work with an implicit public health model dealing with trends in patient populations rather than individual patients. Some stakeholders raised the notion that the Trust processes relating to care planning presume an inherent rationality in situations that are often irrational (e.g. participants detained under the Mental Health Act). There was a perception that there will always be these systematic problems but a good therapeutic relationship can help combat these.

*‘It’s their care, it’s their support, and the relationship – building that relationship is how you involve them in their care. Because they’ve got no power or anything over systems. But they have got power and control over what happened, you know, over how they feel and how they can get better, and how they can relate to somebody’***Key informant 7 (Female, Post-Doctoral Researcher, Academia).**

Key informants acknowledged the role of the broad context in which mental health services were located. They talked about funding cuts and limited resources which made delivering mental health care in services today a daunting task.

*‘Unfortunately at the moment it’s the worst possible time to be trying to challenge it, because I think mental health services are seriously under pressure at the moment. I think there have been times when they [have said] they’re under pressure when I don’t think they were.’***Key informant 12 (Female, Professor, Academia).**

## Discussion

**Fig. 2:**
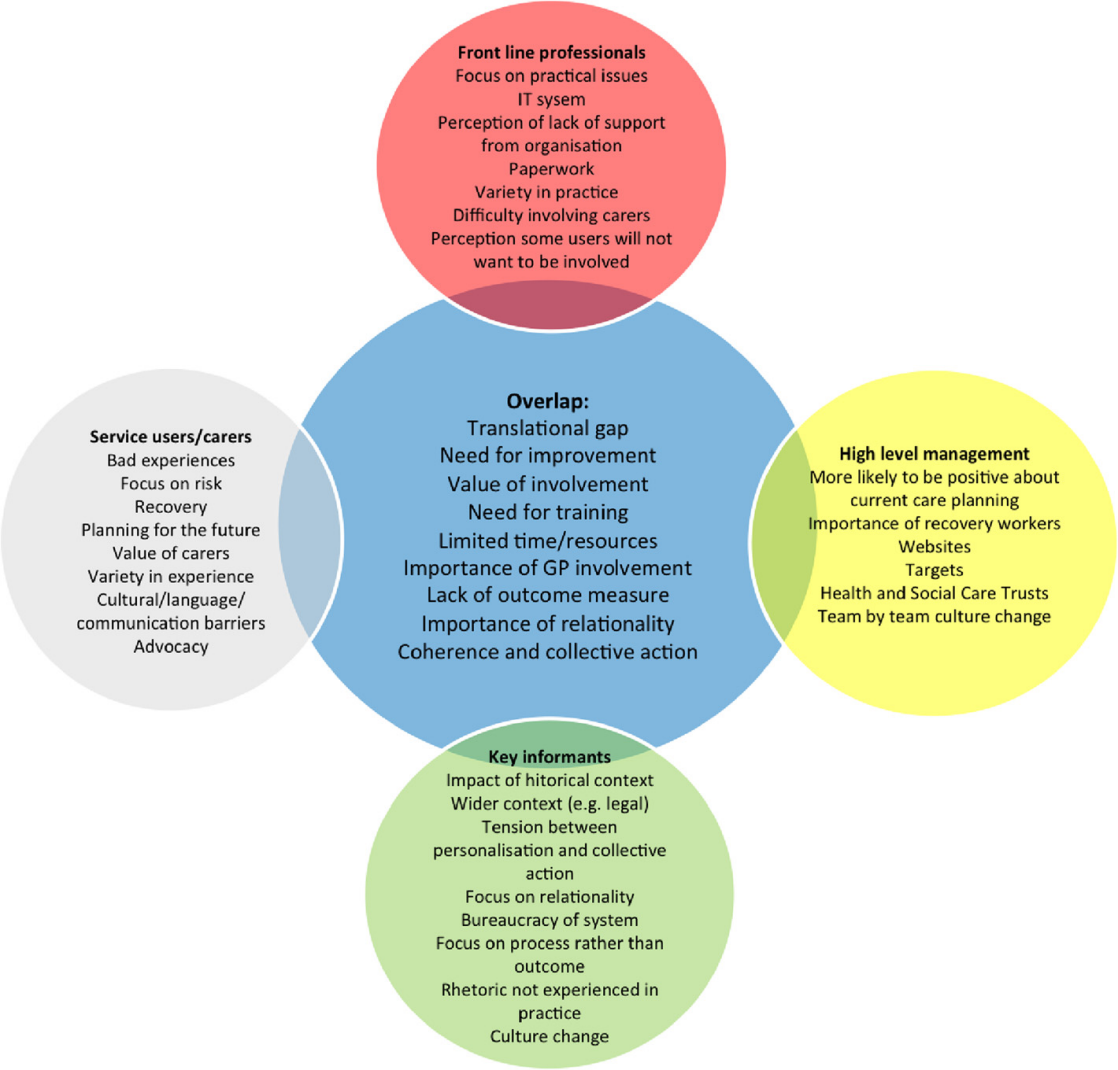
Summary of findings

We conducted a qualitative analysis informed by NPT to explore the potential implementation of a user/carer involved care planning process. Our data supported the data provided by service users, carers and front line professionals, but also included identified additional themes through the incorporation of the perspectives of key stakeholders with an understanding of the meso-level influences of wider political and economic policy related to user involvement in the context of mental health. In the interviews with service users, cares and front line professionals, the identification of outer settings (factors such as the economic, political and social context in which an organisation resides) was much less in evidence than a focus on inner-setting features that needed to be changed. By contrast and in line with other studies [20], the key informants' data illuminated the relevance of structural, political and cultural contexts and described the impact of the historical context of care planning. Figure 2 demonstrates the added value of incorporating the perspectives of these key informants.

The process of implementation involves the need for the evaluation of knowledge at a variety of levels in order to ascertain what is likely to happen both at the design stage of a proposed intervention and long before it becomes used routinely [21]. The findings of this study help locate and identify elements of the current care planning context and process in the mental health field that are likely to impact on the implementability of user-focussed care planning.

Interviews with key informants, analysed and sensitised using implementation theory, revealed facets that need to be considered in the training and its implementation in order to influence the likelihood of adoption. Data collected as part of this study confirmed the relevance of flagging up likely aspects of implementation which could usefully be considered prior to designing the training intervention as part of the logic that upstream considerations about downstream implementation are more likely to promote the normalisation of an intervention in practice. Narrative accounts suggested that a strategic focus on a broader aspirational training programme, with the possibility of implementation across a range of mental health services, would be beneficial in preference to focussing on implementation in one or two sites.

At the level of ideology, the spread of this sort of care planning innovation was viewed as timely. Timeliness is key to spreading and sustaining new practices and in this respect the spread of this sort of care planning innovation was viewed in the context of the battle to change hearts and minds to move towards user/carer orientated planning as having been won [22]. Stakeholders considered that arguments for the need to improve involvement in care planning would receive sufficient traction from practitioners on the ground given the acceptance and embeddedness of a logic of care of user involvement which characterised considerations of mental health practices more generally. Potential problems which may arise were seen as coming from attempts at implementation at the meso and organisational levels of mental health settings, rather than at the interface between users and health professionals [23]. At a systemic level, the dominance of irrelevant or dysfunctional recording processes and the lack of ability to do these efficiently were viewed as taking precedence over the competing imperatives to ensure personalisation and moving beyond a tick box approach to care planning. A further barrier was the prioritisation of outcome over process in terms of organisational imperatives. These issues would need to be considered alongside evidence that suggests that individual practitioner skills also need to be developed [9].

Interviews with key informants supported the imperative of a focus on relationality and relationships and demonstrated how these could be eroded through attempts at practice standardisation (e.g. policy and targets) as well as within the current climate of limited resources. However, where good relationships pervade, despite these issues, they have the potential to counteract systemic problems inherent within mental health trusts (e.g. power imbalance, stigma, medical hierarchy and limited resources) [24]. These points flag the desirability of a focus on developing a specific outcome measure related to involvement and relationality in care planning which transcends more traditionally stated policy and targets.

Interviews with key informants illuminated the structural, political and cultural contexts of services and described the impact of the historical context of care planning. Given their distance from services, participants could comment critically on cultural issues within services. Central to accounts from key informants was a tension between personalisation agendas and the collective action associated with mental health service users and the bureaucratic nature of services, which results in policies and procedures that assume individual rationality in situations that are not themselves rational.

Previous research has alluded to senior management distance from front line services which results in a lack of understanding of the difficulties in providing services at the interface with users [25, 26]. This view was supported by key informants in this study who pointed to the use of rhetoric within services by high level management. This resonated with interviews undertaken with senior managers which demonstrated that whilst they had sympathy and some understanding of the issues raised by service users and professionals they were more likely to describe current service provision in a more positive light than those who experienced it on a day to day basis [4, 5, 8, 9].

Finally, the commitment of staff to the organisation – an important element in implementation process – was felt to include an implicit modicum of cynicism or lack of conviction about the extent and nature of organisational support to see through the changes required. In short, buy-in to user involvement is evident and committed but the capacity of the organisation to support this seemed less convincing. This suggests the need for the active involvement of higher level management in training and delivery who were often seen to be too far removed from front line services to understand current issues.

### Strengths and weaknesses of the current study

The strength of this study lies in its use of qualitative interviews combined with the Normalisation Process Theory as a sensitising tool which allowed for the detailed solicitation of the views of key informants in relation to the barriers and facilitators to the implementation of a user/carer led training programme to improve involvement in care planning. The sample size of 13 is relatively small considering the target population. However, we are confident that the data collected were sufficient to enable a thorough consideration of the potential implementation issues, given that they were contextualised with data provided from a large number of service users, carers and front line professionals. The analysis was also combined with reports from a mapping exercise from each host organisation.

This paper demonstrates how the inclusion of key informants more distanced from front line services can complement and expand upon the views of other key groups (e.g. service users, carers and front line professionals). The interviews asked participants to consider potential implementation issues and those raised will be considered during the course of the trial to test the effectiveness of the training programme.

## Conclusion

This study incorporating the views of key informants collected as part of work for the EQUIP Research programme raised some important issues to consider prior to implementing a training programme for mental health professionals designed to improve service user and carer involvement in care planning. These potential issues relating to implementation of the training package will be used to support the design of the intervention and will also be explored longitudinally in a randomised controlled control within the nested process evaluation.

## Additional files

Additional file 1:
**Interview Schedule.** (DOCX 17 kb) Additional file 2:
**Links between presented themes and NPT components.** (DOCX 16 kb)
