# Supplementary material for: Re-inventing care planning in mental health: stakeholder accounts of the imagined implementation of a user/carer involved intervention
Source: BMC Health Serv Res. 2015 Oct 30;15:490. doi: 10.1186/s12913-015-1154-z (PMC4628327; doi:10.1186/s12913-015-1154-z)
Supplement: Additional file 1: — Interview Schedule. (DOCX 17 kb) [file 12913_2015_1154_MOESM1_ESM.docx]

**Additional file 1: Interview Schedule**

- Introductions; reminder of purpose of study and principles of informed consent; paperwork signed (and countersigned); Dictaphone turned on.

--------------------------------------------------------------------------------------------------------------------------------------

*Experiences and Current Organisational Processes/Systems*

- Can you please tell me a little bit about your role in care planning to date?
  - Probe personal experience or experience in current or previous roles?
- What is the historical context of care planning that you are aware of?
  - Probe specific policies, social movements or other drivers?
- What is the current formulation of care planning expected to do?
  - Does it achieve these aims?
  - How much does it influence the nature of management/care?
  - Examples of good care planning?
  - Examples of bad care planning?

- What is the current care planning ethos in the health service as a whole and locally?
  - Does it have a high profile?
  - Is there leadership support/drive for high quality CP? Why/why not?
  - How does this compare with other systems you know about operating elsewhere (e.g. internationally)?
  - What is the difference between rhetoric and reality?
- How do you think care planning is understood among staff in health services/across different levels of health services?
  - What are current attitudes to care-planning among staff? Why?
- What care planning processes/systems are you aware of within health services?
  - CPA
  - Amigos, RIO (care planning IT systems)
  - Experiences of these?
  - Anything else that influences team/individual behaviour in relation to CP?
- Are there any alternatives to care planning in the NHS?
- Can care planning be made to be more functional if so how?
- What is your experience of working with, or knowledge of, user/carer involvement in policy and service delivery?
  - How well does the current system of care planning work with this ethos?
  - What aspects of policy does care planning fulfil?
    - Probe specific user/carer involvement policy.
- What are the impediments to reform of MH services generally and how do these relate specifically to care planning?
- What would make the biggest difference to improving mental health services currently?

*User/Carer Involvement*

- In the context of care planning – how would you define user involvement?
  - Any examples of user involvement (or lack of)?
  - What has been the impact of this?
- What would you see as being the purpose of a user/carer led care planning approach?
  - What might be the advantages/disadvantages of increasing user/carer involvement?
- How do you think we can best achieve effective or optimal user involvement in CP?
  - Are there some areas where user involvement is easier to achieve?
  - Are there some areas where user involvement is more difficult to achieve?
  - Have you got any examples?
  - What can we learn from this?
- What are the barriers to effective user involvement in care planning?
  - Organisational?
  - Relational?
  - Individual?
- How do you think staff within health services would distinguish between a user/carer led approach and current care planning?
