# Supplementary material for: Re-inventing care planning in mental health: stakeholder accounts of the imagined implementation of a user/carer involved intervention
Source: BMC Health Serv Res. 2015 Oct 30;15:490. doi: 10.1186/s12913-015-1154-z (PMC4628327; doi:10.1186/s12913-015-1154-z)
Supplement: Additional file 2: — Links between presented themes and NPT components. (DOCX 16 kb) [file 12913_2015_1154_MOESM2_ESM.docx]

**Additional file 2: Links between presented themes and NPT components**

**Coherence:**

*‘Shifting ownership’ - Clear understanding about the need for change and optimal conditions for care planning*

*Bridging the translational gap – the impact of bureaucracy and the effect of a long standing agenda of control and coercion within services*

*Rationality within the system: More important than practice innovations?*

**Cognitive participation**

*The value of and work associated with implementing user/carer involved care planning*

*The individual versus the collective and the inhospitable ethos of mental health services*

**Collective action**

*The role of individual differences in relation to the delivery of user carer involved care planning*

*Connection and culture as barriers to implementation*
